# Supplementary figures and images for: Modifying TIMER to generate a slow-folding DsRed derivative for optimal use in quickly-dividing bacteria
Source: PLoS Pathog. 2021 Jul 2;17(7):e1009284. doi: 10.1371/journal.ppat.1009284 (PMC8291646; doi:10.1371/journal.ppat.1009284)

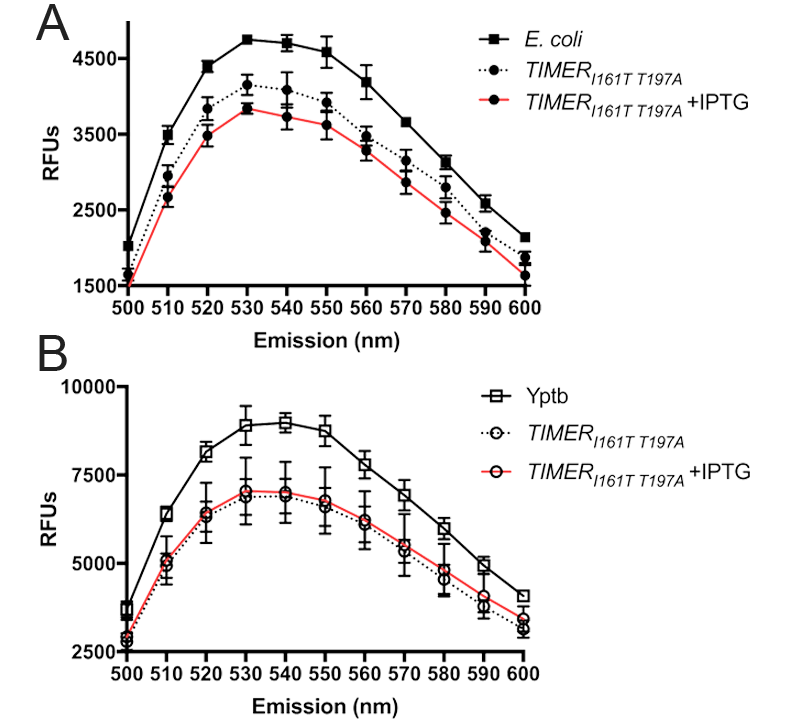

Supplement: S1 Fig — The indicated strains contain the TIMERI161T T197A variant in the low copy vector, pMMB67EH. Strains were grown at 37° C in 96 well plates with shaking for 24h. Fluorescence was detected with a spectral curve with excitation at 480nm and emission detected between 500nm-600nm. Relative fluorescence units (RFUs) represent raw values with media only background subtracted, data represents mean of three biological replicates. A) E. coli strains: E. coli is the non-fluorescent DH5αλpir E. coli parent strain. B) Y. pseudotuberculosis strains: Yptb is the non-fluorescent WT IP2666 parent strain. (TIF) [file ppat.1009284.s001.tif]

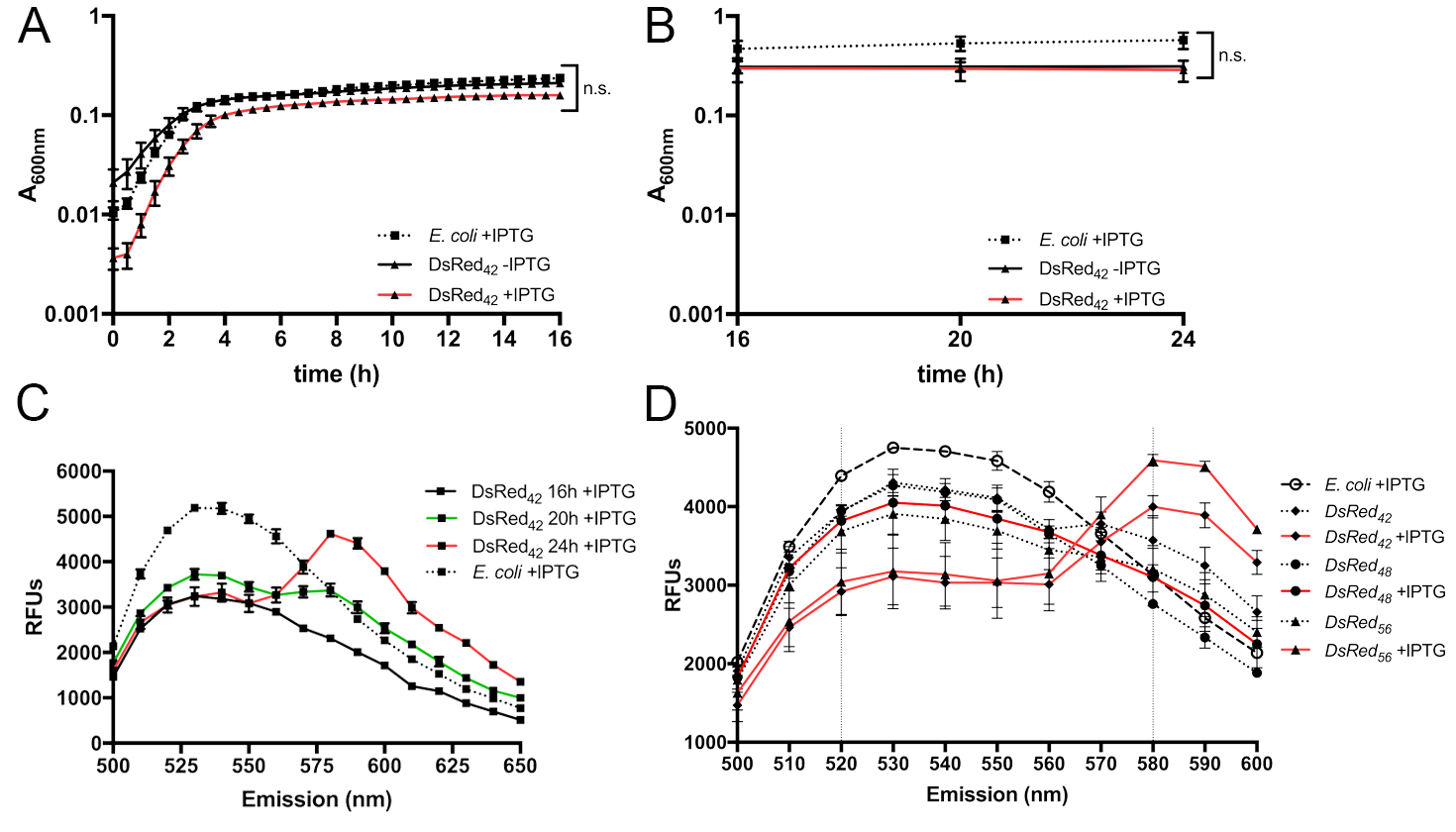

Supplement: S2 Fig — E. coli strains (XLI-BLUE background) contain the indicated DsRed variants, expressed from the high copy pUC18 plasmid. Strains were grown for the indicated times at 37° C in 96 well plates with shaking. Fluorescence was detected with a spectral curve with excitation at 480nm and emission detected between 500nm-600nm. Relative fluorescence units (RFUs) represent raw values with media only background subtracted, data represents mean of three biological replicates. A) Optical density of the indicated strains was measured by absorbance (A600nm) over time (hours, h), during incubation at 37° C in a plate reader. B) The indicated strains were grown as described above, and optical density was measured by absorbance (A600nm) at the indicated timepoints (hours, h) C) DsRed variants were grown alongside the non-fluorescent WT E. coli strain in the presence of IPTG for the indicated times. E. coli +IPTG: 24h growth. D) DsRed variants grown alongside the non-fluorescent WT E. coli strain, in the presence or absence of IPTG for 24h. Vertical dotted lines: 520nm (green fluorescence) and 580nm (red fluorescence). Statistics: A)- B) Two-way ANOVA with Tukey’s multiple comparison test; n.s.: not-significant. (TIF) [file ppat.1009284.s002.tif]

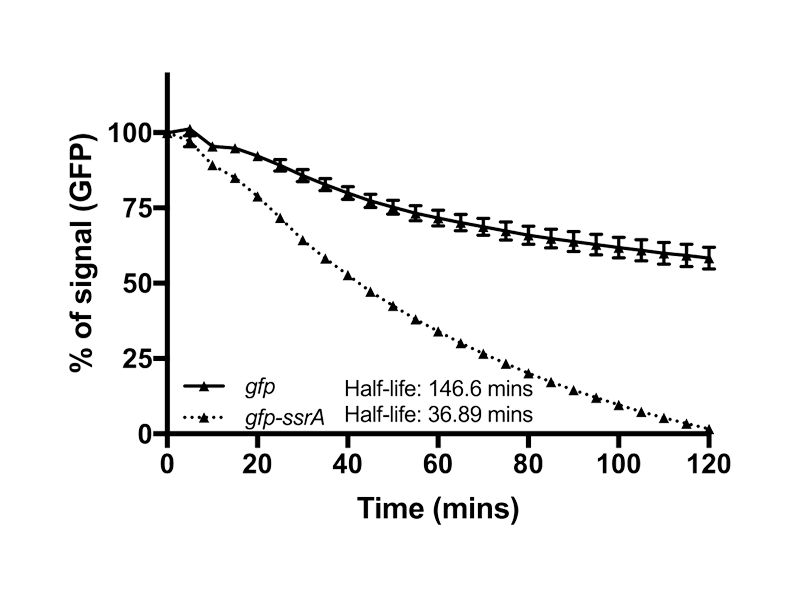

Supplement: S3 Fig — WT IP2666 containing IPTG-inducible gfp or gfp-ssrA were grown overnight (16h) in the presence of IPTG. Cells were pelleted and resuspended in PBS, and kanamycin was added to inhibit additional protein translation. GFP signal was detected with 480nm excitation/520nm emission every 5 minutes for 120 minutes in a plate reader, to detect the half-life of each fluorescent protein by the one-phase exponential decay equation. Fluorescent values are expressed as % of signal (GFP), where time 0 equals 100% signal intensity and 0% signal intensity indicates the limit of detection. Data represents mean of three biological replicates. (TIF) [file ppat.1009284.s003.tif]
